# TGF-β2 downregulates osteogenesis under inflammatory conditions in dental follicle stem cells

**DOI:** 10.1038/s41368-018-0028-8

**Published:** 2018-10-09

**Authors:** Soyoun Um, Joo-Hee Lee, Byoung-Moo Seo

**Affiliations:** 1 Biotooth Engineering Lab, Dental Research Institute, Dental Regenerative Biotechnology, Department of Dental Science, School of Dentistry, Seoul National University, Seoul, Korea; 2 Biotooth Engineering Lab, Department of Oral and Maxillofacial Surgery and Craniomaxillofacial Life Science, Dental Research Institute, School of Dentistry, Seoul National University, Seoul, Korea

## Abstract

Bone formation is important for the reconstruction of bone-related structures in areas that have been damaged by inflammation. Inflammatory conditions such as those that occur in patients with rheumatoid arthritis, cystic fibrosis, and periodontitis have been shown to inhibit osteoblastic differentiation. This study focussed on dental follicle stem cells (DFSCs), which are found in developing tooth germ and participate in the reconstruction of alveolar bone and periodontal tissue in periodontal disease. After bacterial infection of inflamed dental tissue, the destruction of bone was observed. Currently, little is known about the relationship between the inflammatory environment and bone formation. Osteogenic differentiation of inflamed DFSCs resulted in decreased alkaline phosphatase (ALP) activity and alizarin red S staining compared to normal DFSCs. Additionally, in vivo transplantation of inflamed and normal DFSCs demonstrated severe impairment of osteogenesis by inflamed DFSCs. Protein profile analysis via liquid chromatography coupled with tandem mass spectrometry was performed to analyse the differences in protein expression in inflamed and normal tissue. Comparison of inflamed and normal DFSCs showed significant changes in the level of expression of transforming growth factor (TGF)-β2. *Porphyromonas gingivalis* (*P.g*.)-derived lipopolysaccharide (LPS) was used to create in vitro inflammatory conditions similar to periodontitis. The osteogenic differentiation of LPS-treated DFSCs was suppressed, and the cells displayed low levels of TGF-β1 and high levels of TGF-β2. DFSCs treated with TGF-β2 inhibitors showed significant increases in alizarin red S staining and ALP activity. TGF-β1 expression was also increased after inhibition of TGF-β2. By examining inflamed DFSCs and LPS-triggered DFSCs, these studies showed both clinically and experimentally that the increase in TGF-β2 levels that occurs under inflammatory conditions inhibits bone formation.

## Osteogenesis: Transforming growth factor-β2 inhibits bone formation during inflammation

During inflammation, increased transforming growth factor (TGF)-β2 inhibits bone formation in dental follicle stem cells (DFSCs). Hitherto, the relationship between inflammation and bone formation has been poorly understood. But a team headed by Byoung-Moo Seo of Seoul National University, Republic of Korea examined the different functions of two types of TGF-β (a protein that is a key regulator of bone formation): TGF-β1 and TGF-β2. By means of cell cultures and in vivo experiments in mice, the team conducted its investigation on DFSCs: stem cells (non-specialised cells) in the dental follicle, which surrounds a tooth before it erupts. The authors found that inflammation led to an increase in TGF-β2, and that increase inhibited bone formation. The results of the study have implications for the future therapeutic application of DFSCs in bone-loss diseases.

## Introduction

Bone regeneration is critical for reconstructing bone structures that have been destroyed by pathological processes or trauma. In general, mesenchymal stem cells are recruited to the site of destruction, where they participate in the regeneration of damaged bone. However, bone formation in damaged areas is influenced by the local inflammatory conditions and the degree of tissue injury. Bone-related diseases induce an inflammatory environment that might inhibit osteoblastic differentiation and bone formation; this has been demonstrated in rheumatoid arthritis, cystic fibrosis, and periodontitis.^1,2^

Transforming growth factor (TGF)-β is not only a critical regulator of osteogenic differentiation, which it regulates by activating downstream Smad signalling pathways,^3,4^ but also acts as an immunoregulatory cytokine.^5–7^ However, conflicting results regarding the effects of TGF-β on bone formation have been reported. TGF-β controls bone formation by modulating osteoblastic cell proliferation and differentiation in vitro and by increasing the number of osteoblasts in vivo. TGF-β also regulates inflammation by suppressing the production of pro-inflammatory cytokines.^8,9^ In contrast, TGF-β was shown to suppress osteogenesis in murine cell lines and human mesenchymal stem cells in vitro.^10–12^ These controversial results might be related to the indiscriminate use of TGF-β1 and TGF-β2. In this work, we focussed on the distinct functions of TGF-β1 and TGF-β2 in osteogenesis and under inflammatory conditions.

Exogenous TGF-β2 reduces bone and cartilage formation in healing fractures in rabbits.^13^ Comparison of the patterns of expression of TGF-β1 and TGF-β2 in human bone samples showed that, in contrast to TGF-β1, TGF-β2 levels were highly enhanced in osteoarthritic bone compared to normal bone.^14^ These results suggest that TGF-β2 may have a distinct role in bone formation under inflammatory conditions.

Smad signalling, which is activated by TGF-β binding to TGF-β receptors, results in the inhibition of inflammatory cytokine production by immune cells.^15,16^ Additionally, Smad2-deficient or Smad3-deficient mice exhibit inflammatory disease, suggesting that both Smad2 and Samd3 regulate immune reactions.^17–20^ Overall, Smad2/3 signalling is important in both bone formation and inflammation.

Dental follicles, which originate from ectomesenchymal cells, surround the developing tooth germ and contain the periodontal precursor cells that give rise to periodontal tissue consisting of cementum, periodontal ligaments, and alveolar bone during tooth development.^21–23^ Recently, mesenchymal stem cells (MSCs) from dental follicles were isolated and differentiated into clonogenic, plastic-adherent, fibroblast-like cells.^24,25^ The multipotency of dental follicle stem cells (DFSCs) makes these cells potentially useful in the reconstruction of bone under inflammatory conditions.^26–29^ As a disease that is frequently associated with tooth loss, periodontal disease is defined as the destruction of alveolar bone and periodontal tissues by bacterial infection.^26,27,30^ Therapeutic strategies for the treatment of periodontitis include not only the control of local inflammation but also the regeneration of new periodontal tissues attached to the surface of the tooth root.

*Porphyromonas gingivalis* (*P.g*.) is a Gram-negative bacterium that is frequently found in oral tissue. *P.g*.-derived lipopolysaccharide (LPS) is a crucial virulence factor that is aetiologically associated with the initiation and development of periodontal disease.^31,32^ It has been reported that *P.g*.-derived LPS acts as a potent stimulator of inflammatory cytokine production and bone resorption.^33^ However, the exact mechanisms and relationships between infectious disease and bone formation are unclear. At present, little is known about the relationship between the inflammatory environment and downregulation of bone formation. A previous study showed that the toll-like receptors (TLR) TLR2 and TLR4 are stimulated by LPS and that LPS triggers cell viability and cytokine secretion by MSCs.^34^ Additionally, direct contact with bacterial toxins on MSCs triggers immune responses. Patients with periodontitis have symptoms such as gingival bleeding and progressive alveolar bone destruction. Therefore, it is necessary to clarify the interrelationship between bone formation and inflammation.

The aim of this study was to investigate the interactions of DFSCs with periodontal pathogens and to thereby demonstrate the relationship between inflammatory conditions and osteogenesis. Unlike TGF-β1, the role of TGF-β2 in the control of osteogenic differentiation has not been defined. We investigated the relationship of TGF-β2 to inflammation and bone regeneration by cross-activating Smad2/3 in DFSCs. We hypothesise that increased TGF-β2 levels caused by inflammation prevent bone formation by DFSCs.

## Results

### Proliferation and osteogenic differentiation of normal and inflamed dental follicle stem cells

**Fig. 1:**
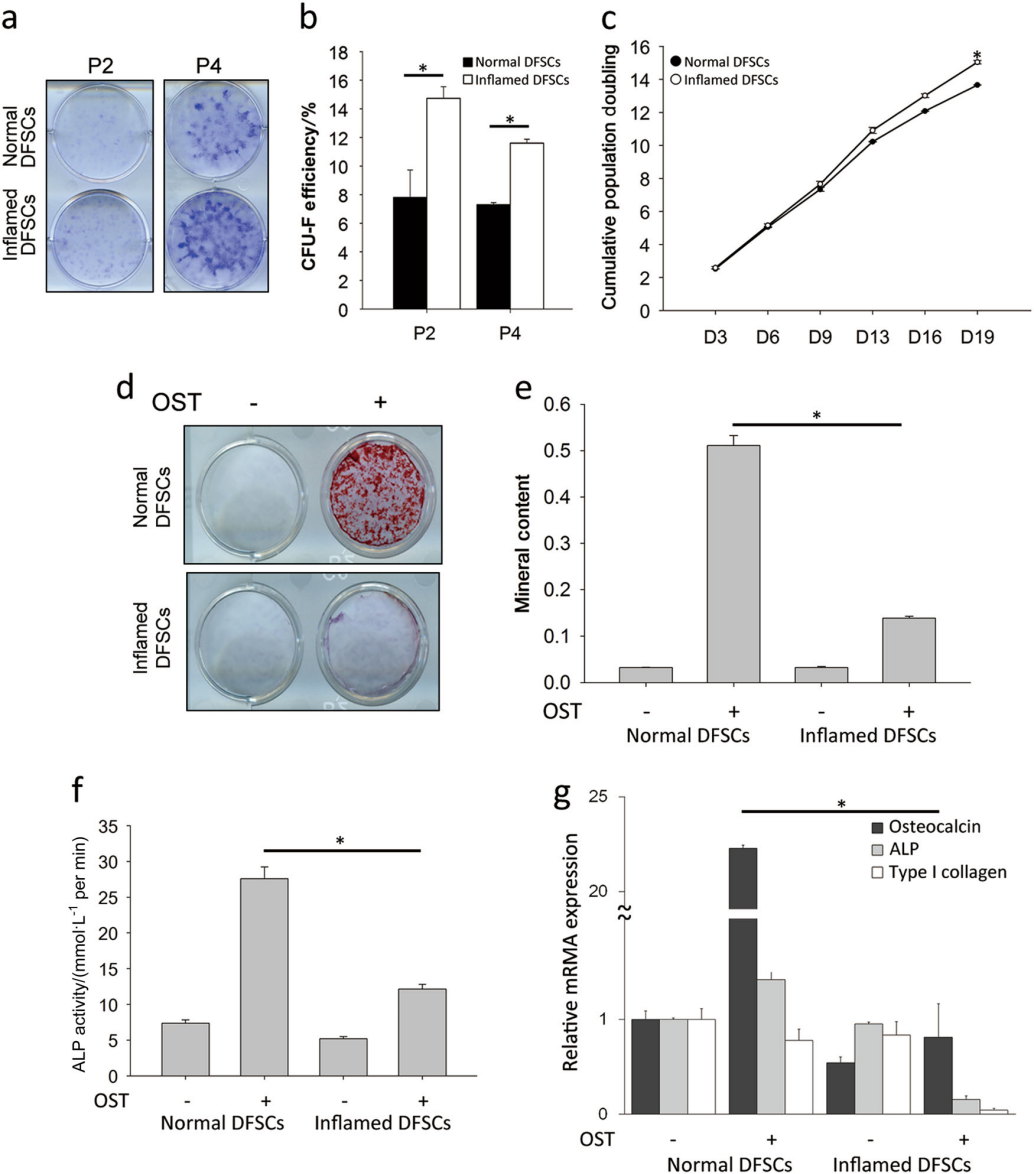
Proliferation and osteogenic differentiation of normal and inflamed dental follicle stem cells (DFSCs). **a** DFSCs isolated from normal and inflamed dental follicles were cultured in normal growth medium. Colony-forming assays using 1% crystal violet were performed at passages 2 and 4. **b** Colonies consisting of more than 50 cells were counted and calculated as a percentage of the number of seeded cells at passages 2 and 4. Inflamed DFSCs showed a higher rate of proliferation. **c** The cumulative population doubling times of normal and inflamed DFSCs were determined by counting the cells on day 3, 6, 9, 13, 16 and 19. There was a significant difference in the rate of proliferation of normal and inflamed DFSCs at day 19. **d** Calcium deposits formed by normal and inflamed DFSCs on osteogenic differentiation were stained using 40 mmol^.^L^-1^ alizarin red S solution on day 21. **e** The mineral contents dissolved by stained calcium deposits were dissolved in 20% methanol and 10% acetic acid. **f** ALP activity, an early marker of osteogenic differentiation, was measured on day 7. **g** Real-time PCR showed that the gene expression of osteocalcin, an osteogenic marker, was downregulated in inflamed DFSCs. The data are presented as the mean ± SD. * *P* < 0.05 (*n* = 3)

Isolated DFSCs from normal and inflamed dental follicle tissues were cultured in stem cell growth medium. As shown in Fig. 1a, b, the colony-forming efficiency (CFE) of inflamed DFSCs (14.7% ± 0.81%) was significantly higher (*P* < 0.05) than that of normal DFSCs (7.8% ± 1.9%) at passage 2. The CFE of inflamed DFSCs was also higher than that of normal DFSCs at passage 4. The DFSCs in the two groups showed similar growth kinetics during expansion through passage 9. After passage 13, an appreciable increase in the number of population doublings of inflamed DFSCs was observed, indicating that the proliferative potential of inflamed DFSCs was higher than that of normal DFSCs (Fig. 1c). Thus, inflamed DFSCs had higher proliferation potential than did normal DFSCs.

To evaluate the effects of inflammation on the osteogenic differentiation of normal and inflamed DFSCs, alizarin red S staining (Fig. 1d, e) and ALP activity (Fig. 1f) were measured. ALP activity, as an early marker of osteogenic differentiation, was assessed on day 7. The ALP activity of normal DFSCs increased significantly by approximately 2.5-fold compared to that of inflamed DFSCs. Similarly, more calcium deposits were observed in normal DFSCs than in inflamed DFSCs. Based on quantification of the mineral contents of the cultures, inflamed DFSCs showed approximately five-fold lower amounts of calcium deposits than normal DFSCs. Osteocalcin expression was also significantly lower in inflamed DFSCs than in normal DFSCs (Fig. 1g). However, ALP and type 1 collagen expression did not differ significantly at the mRNA level. Thus, the osteogenic differentiation of normal and inflamed DFSCs showed significant differences.

### In vivo transplantation of normal and inflamed dental follicle stem cells

**Fig. 2:**
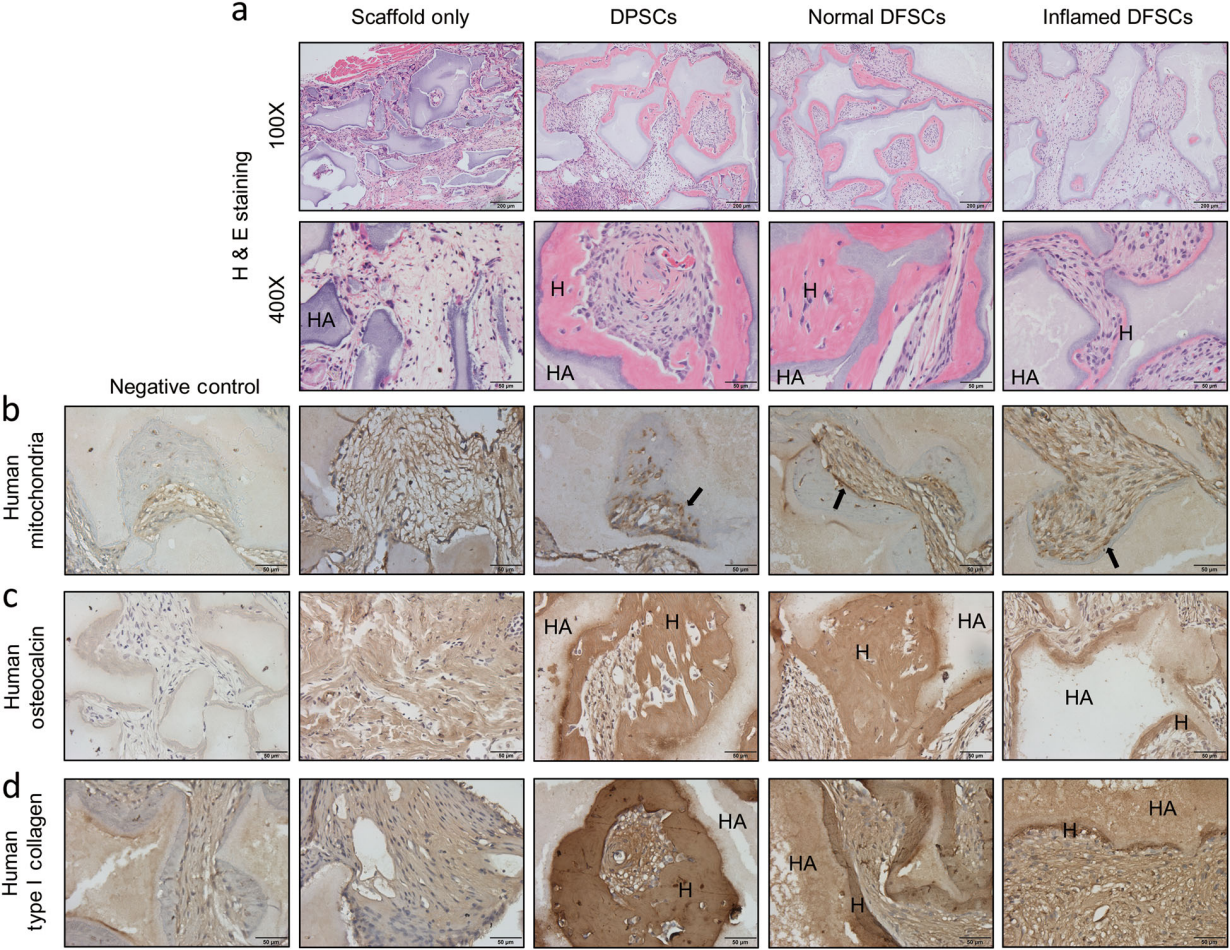
In vivo transplantation of normal and inflamed DFSCs. **a** H&E staining was performed to determine the amount of hard tissue formed by transplanted normal and inflamed DFSCs with HA/TCP after 8 weeks transplantation to the dorsal skin of nude mice. Inflamed DFSCs showed significantly less hard tissue formation. **b** Dense collections of human mitochondria were detected in areas of hard tissue formation. **c**, **d** Hard tissue regions were positively stained for human osteocalcin and type 1 collagen to confirm hard tissue formation by transplanted normal and inflamed DFSCs. The two groups showed significantly different amounts of bone formation

The decreased patterns of osteogenesis observed in vitro were confirmed by assessing hard tissue formation after in vivo transplantation of DFSCs to the backs of nude mice in the presence of HA-TCP carriers for 8 weeks. Dental pulp stem cells (DPSCs) were used as a positive control. In these experiments, the formation of dentin-pulp complex-like mineralised tissue by DPSCs was measured. DFSCs showed the formation of bone-like hard or mineralised tissue. Comparison of hard tissue formation after H&E staining showed that the transplants containing inflamed DFSCs displayed less hard tissue formation than transplants containing normal DFSCs (Fig. 2a). Immunohistochemistry using an antibody against human mitochondria was performed to confirm that the transplanted cells were human DFSCs (Fig. 2b). The hard tissue in the culture was positively stained by antibodies to osteocalcin and type 1 collagen (Fig. 2c, d). These results indicate that hard tissue was formed by both normal and inflamed human DFSCs. Thus, both the in vitro and the in vivo transplantation results showed that inflamed DFSCs display lower levels of osteogenic differentiation.

### Identification of proteins in normal and inflamed dental follicle stem cells

**Fig. 3:**
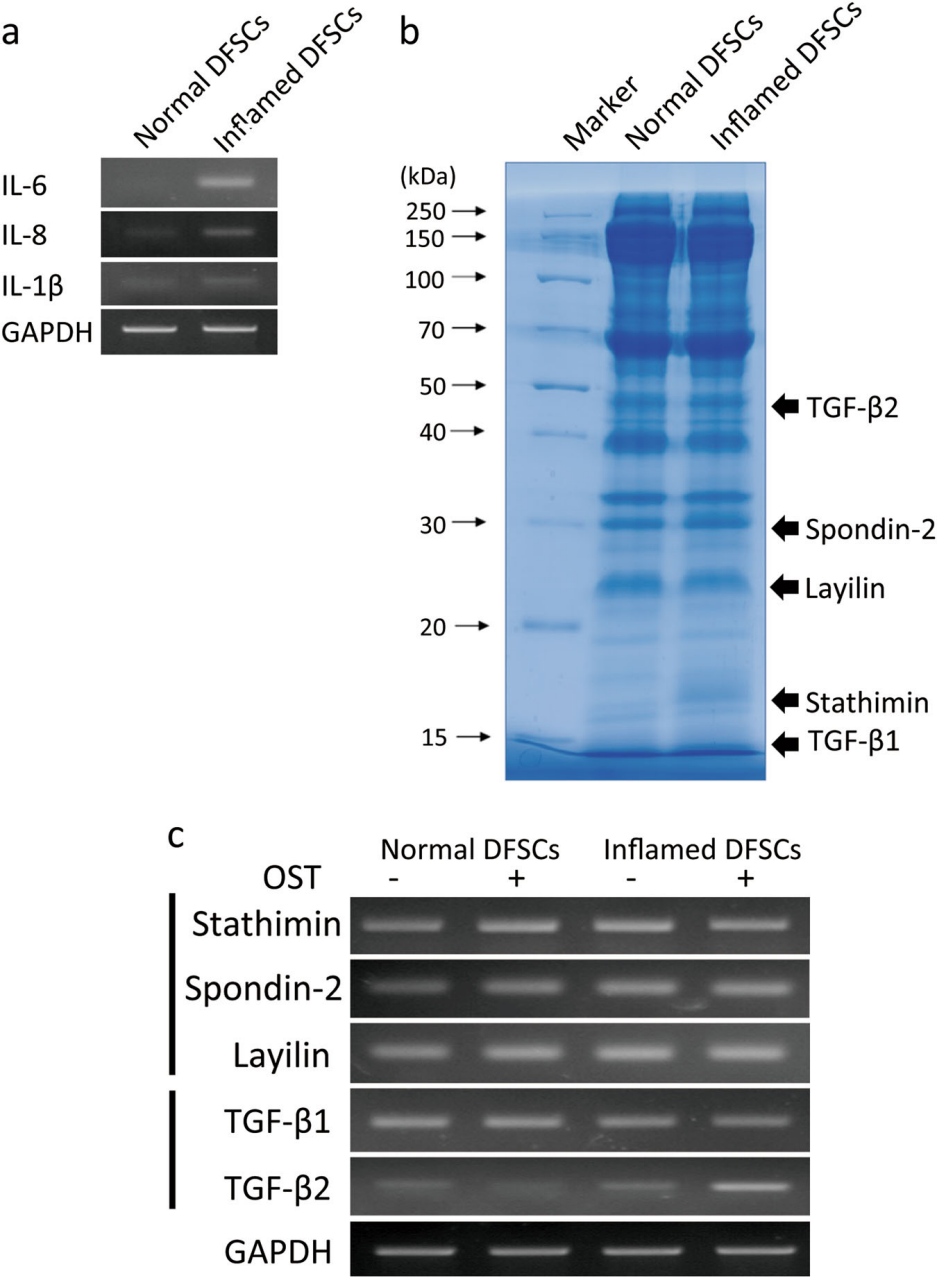
Gene profiling by RT-PCR. **a** The levels of the pro-inflammatory cytokines IL-6, IL-8 and IL-1β in DFSCs isolated from inflamed tissue were measured. **b** From the set of proteins with Mascot scores greater than 30 selected from LC-MS/MS analysis of normal and inflamed DFSCs, proteins related to osteogenic differentiation were selected. The level of expression of the genes encoding these proteins in normal and inflamed DFSCs during osteogenesis was determined by RT-PCR using specific primers. Differences in TGF-β2 gene expression in normal and inflamed DFSCs during osteogenesis were detected

RT-PCR was conducted to assess the expression of pro-inflammatory cytokines such as IL-6, IL-8 and IL-1β by inflamed DFSCs (Fig. 3a). To evaluate the differences between normal and inflamed DFSCs, protein profile analysis followed by liquid chromatography coupled with tandem mass spectrometry (LC-MS/MS) was performed. Proteins were extracted from the cells, harvested by centrifugation and separated using 1-dimensional SDS-PAGE (Fig. 3b). Considerably different concentrations of protein were found in inflamed DF and normal DF samples, especially in the case of proteins with molecular weights of approximately 15, 17, 25, 30 and 50 kDa. These bands were identified by LC-MS/MS and analysed using Mascot engines (Table 1). Of the proteins with Mascot scores greater than 30, proteins related to osteogenesis, proliferation, and inflammation were selected. The expression of stathimin, spondin-2, and layilin genes was enhanced in inflamed DFSCs compared to DFSCs cultured under normal conditions (Fig. 3c). Whereas expression of the TGF-β1 gene in normal and inflamed DFSCs did not differ, TGF-β2 expression by inflamed DFSCs was slightly increased compared to its expression in normal DFSCs. On the other hand, osteogenic conditions influenced the expression of TGF-β1 and TGF-β2 differently. Expression of the TGF-β2 gene in inflamed DFSCs was significantly increased under osteogenic conditions. Thus, the results from both protein profiling analysis and RT-PCR showed that inflamed DFSCs display increased TGF-β2 gene expression during osteogenic differentiation (Table 1; Fig. 3c).

**Table 1:** Mass spectrometry analysis by comparison of normal and inflamed dental follicle in different bands of 1D-SDS-PAGE gel

Sample	Number	NCBI BLAST	Protein name	MASCOT score	Mass/Da
Normal DF	1	GI:5821385	MTH1a (p26) (Homo sapiens)	95	22,537
	2	GI:82571735	PPIF protein (Homo sapiens)	71	16,530
	3	GI:284164	Arginine-rich protein—human	63	26,889
	4	GI:5031851	Stathmin isoform a (Homo sapiens)	59	17,292
	5	GI:223480	Dismutase, Cu/Zn superoxide	53	15,792
	6	GI:18490199	TWF1 protein (Homo sapiens)	50	28,805
	7	GI:385719190	Layilin isoform 3 (Homo sapiens)	38	25,555
	8	GI:5453678	Epididymal secretory protein E1 precursor (Homo sapiens)	38	16,559
	9	GI:30506	Desmoglein type 1 [Homo sapiens)	32	113,644
	10	GI:9502027	Nucleotide binding protein (Homo sapiens)	27	30,204
	11	GI:57997547	Hypothetical protein (Homo sapiens)	37	13,865
	12	GI:3318841	Chain A, Horf6 A novel human peroxidase enzyme	34	25,011
	13	GI:4503987	Gamma-glutamyl hydrolase precursor (Homo sapiens)	77	35,941
	14	GI:31291	Unnamed protein product (Homo sapiens)	70	38,589
	15	GI:34234	Laminin-binding protein (Homo sapiens)	55	31,774
	16	GI:16549132	Unnamed protein product (Homo sapiens)	54	60,904
	17	GI:6912682	Spondin-2 precursor (Homo sapiens)	40	35,750
	18	GI:49256867	RAD50 protein, partial (Homo sapiens)	29	84,003
	19	GI:18376667	hSSH-2A (Homo sapiens)	27	21,650
Inflamed DF	1	GI:36038	rho GDP dissociation inhibitor (GDI) (Homo sapiens)	54	23,179
	2	GI:4808278	lanosterol synthase (Homo sapiens)	33	83,225
	3	GI:4506193	Proteasome subunit beta type-1 (Homo sapiens)	137	26,472
	4	GI:4506181	Proteasome subunit alpha type-2 (Homo sapiens)	89	25,882
	5	GI:21465651	Chain J, Crystal structure of the mammalian 20 s proteasome at 2.75A resolution	84	22,915
	6	GI:31543380	Protein DJ-1 (Homo sapiens)	81	19,878
	7	GI:4506243	Polypyrimidine tract-binding protein 1 isoform a (Homo sapiens)	79	59,596
	8	GI:4504447	Heterogeneous nuclear ribonucleoproteins A2/B1 isoform A2 (Homo sapiens)	69	35,984
	9	GI:348239	Unnamed protein product (Homo sapiens)	50	54,233
	10	GI:178775	Proapolipoprotein, partial (Homo sapiens)	44	28,944
	11	GI:179462	N-acetyl-beta-glucosaminidase prepro-polypeptide, partial (Homo sapiens)	44	64,321
	12	GI:31189	Unnamed protein product (Homo sapiens)	36	23,182
	13	GI:182399	Farnesyl pyrophosphate synthetase (EC 2.5.1.1) (Hoo sapiens)	64	40,495
	14	GI:557563	Transforming growth factor beta 2 (Homo sapiens)	41	47,603
	15	GI:3294548	Cathepsin Z precursor (Homo sapiens)	40	33,860
	16	GI:33431109	Transforming growth factor beta 1 (Homo sapiens)	39	12908
	17	GI:10798804	Sperm antigen (Homo sapiens)	29	84,869

Proteins identified by LC-MS/MS (liquid chromatography-coupled electrospray ionisation MS/MS) were searched against the NCBI database using the MASCOT search software. The list includes all significant hits

### Mimicking of the inflammatory environment by treatment of DFSCs with *P.g.*-derived LPS

**Fig. 4:**
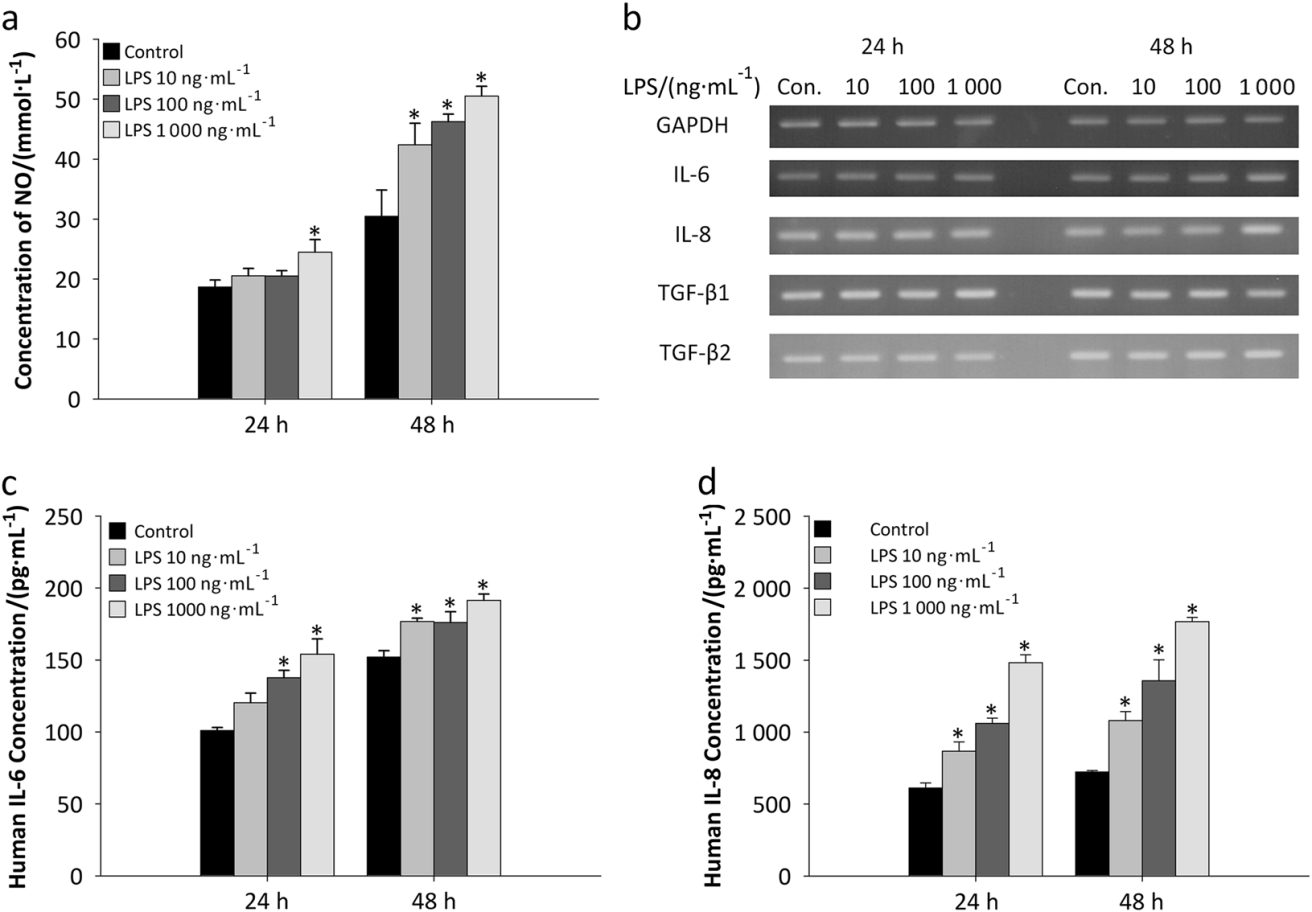
*Porphyromonas gingivalis* (*P.g*.)-derived LPS-induced inflammation mimics inflammatory conditions in DFSCs. **a** Cultured DFSCs were treated with various concentrations of LPS (10, 100 and 1000 ng**·**mL^−1^) and allowed to secrete nitric oxide (NO) for 24 and 48 h. **b** The levels of the pro-inflammatory cytokines IL-6 and IL-8 were increased by treatment with 1000 ng**·**mL^−1^ LPS for 48 h. However, there was no significant difference in the gene expression of TGF-β1 and TGF-β2 in cells maintained in conditional medium with LPS treatment. **c**, **d** The protein levels of IL-6 and IL-8 were also increased after LPS treatment. The data are presented as the mean ± SD. * *P* < 0.05 (*n* = 3)

To mimic the inflammatory environment, LPS treatment was used. When normal DFSCs were treated with *P*.*g*.-derived LPS at 10, 100 and 1 000 ng**·**mL^−1^ for 24 and 48 h, the concentrations of nitric oxide (NO) in the cell culture supernatants increased (Fig. 4a). LPS-stimulated NO production by DFSCs was significantly increased by treatment of the cells with 100 and 1000 ng**·**mL^−1^ LPS. To verify that *P.g*.-derived LPS promoted an inflammatory environment, the expression of the pro-inflammatory cytokines IL-6 and IL-8 was measured by RT-PCR (Fig. 4b). The results indicated that treatment of the cells with 1000 ng**·**mL^−1^ LPS for 48 h provoked expression of both the IL-6 and the IL-8 genes. A slight decrease in the expression of the TGF-β1 gene was also observed after 48 h of LPS treatment. However, TGF-β2 expression in cells maintained in normal medium did not change after LPS treatment. The ELISA results also confirmed that LPS treatment mimicked the effects of an inflammatory environment on DFSCs with respect to the secretion of IL-6 (Fig. 4c) and IL-8 (Fig. 4d). These results show that *P.g*.-derived LPS created inflammatory conditions for DFSCs and caused them to resemble inflamed DFSCs.

### Effect of LPS on the proliferation and osteogenic differentiation of DFSCs

**Fig. 5:**
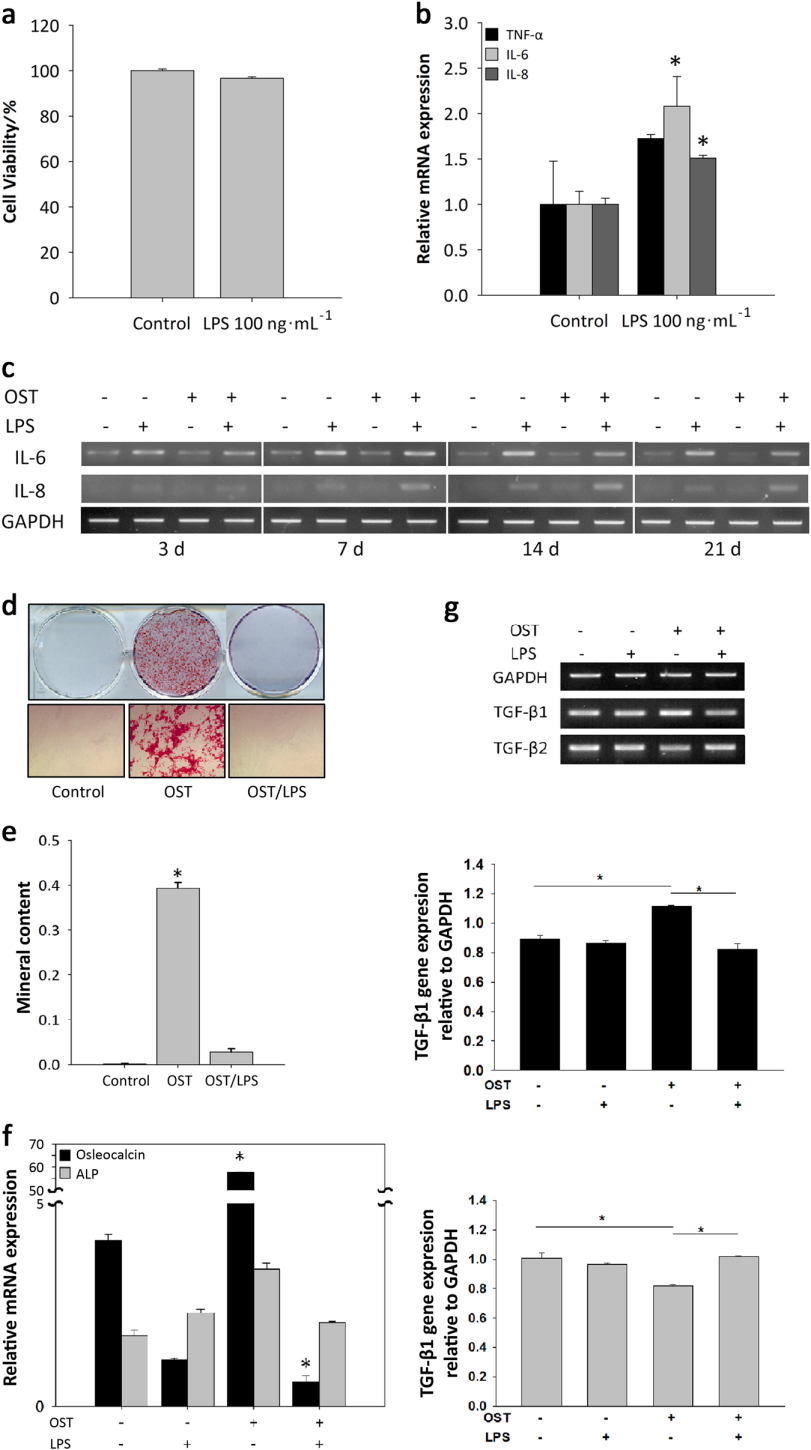
Downregulation of the osteogenic differentiation of DFSCs after exposure to *P.g.-*derived LPS. **a** MTT assays were performed to determine cell viability after LPS treatment. LPS at 100 ng**·**mL^−1^ had no effect on cell viability. **b** Real-time PCR showed that the pro-inflammatory cytokines IL-6 and IL-8 were secreted after treatment of cells with 100 ng**·**mL^−1^ LPS treatment in conditional medium. **c** IL-6 and IL-8 were also expressed during the osteogenic differentiation of cells treated with 100 ng**·**mL^−1^LPS. **d**, **e** Calcium deposition during osteogenesis was inhibited by 100 ng**·**mL^−1^ LPS treatment. The dissolved mineral content of the medium was decreased approximately 4.5-fold compared to the control without LPS treatment. **f** Osteocalcin gene expression was significantly inhibited. **g** Comparisons of TGF-β1 and TGF-β2 gene expression by RT-PCR were performed after differentiating osteogenic tissue in the presence of 100 ng**·**mL^−1^ LPS for 2 weeks. During osteogenesis, TGF-β1 expression increased significantly, whereas TGF-β2 showed decreased expression. During LPS treatment, TGF-β1 and TGF-β2 expression changed in an inverse manner. LPS triggered higher TGF-β2 expression during osteogenesis. The data are presented as the mean ± SD. * *P* < 0.05 (*n* = 3)

To determine the effects of inflammation on the proliferation and osteogenesis of DFSCs, DFSCs were stimulated with LPS during osteogenesis. Treatment with LPS at 100 ng**·**mL^−1^ did not affect cell viability (Fig. 5a). However, the pro-inflammatory cytokines IL-6 and IL-8 were secreted by DFSCs at significantly different levels than under normal conditions (Fig. 5b). The secretion of these inflammatory cytokines was maintained during osteogenesis (Fig. 5c). At the early stage of osteogenic differentiation, IL-6 and IL-8 were expressed. The inflammatory environment triggered by LPS also resulted in suppression of calcium deposit formation by DFSCs (Fig. 5d, e). Similar to alizarin red S staining, osteocalcin expression was significantly decreased by approximately 55-fold after LPS treatment compared to the control with no LPS treatment (Fig. 5f). Interestingly, the TGF-β1 gene was highly expressed during osteogenesis, whereas TGF-β1 expression was significantly suppressed in LPS-treated cells during osteogenic differentiation. In addition, TGF-β2 levels decreased significantly during osteogenic differentiation, whereas LPS treatment of DFSCs undergoing osteogenic differentiation triggered the expression of TGF-β2 (Fig. 5g). Taken together, these results demonstrate that LPS treatment of DFSCs mimicked the inflammatory environment, creating an environment similar to that of inflamed DFSCs.

### The effects of TGF-β2 on LPS-stimulated osteogenic differentiation

**Fig. 6:**
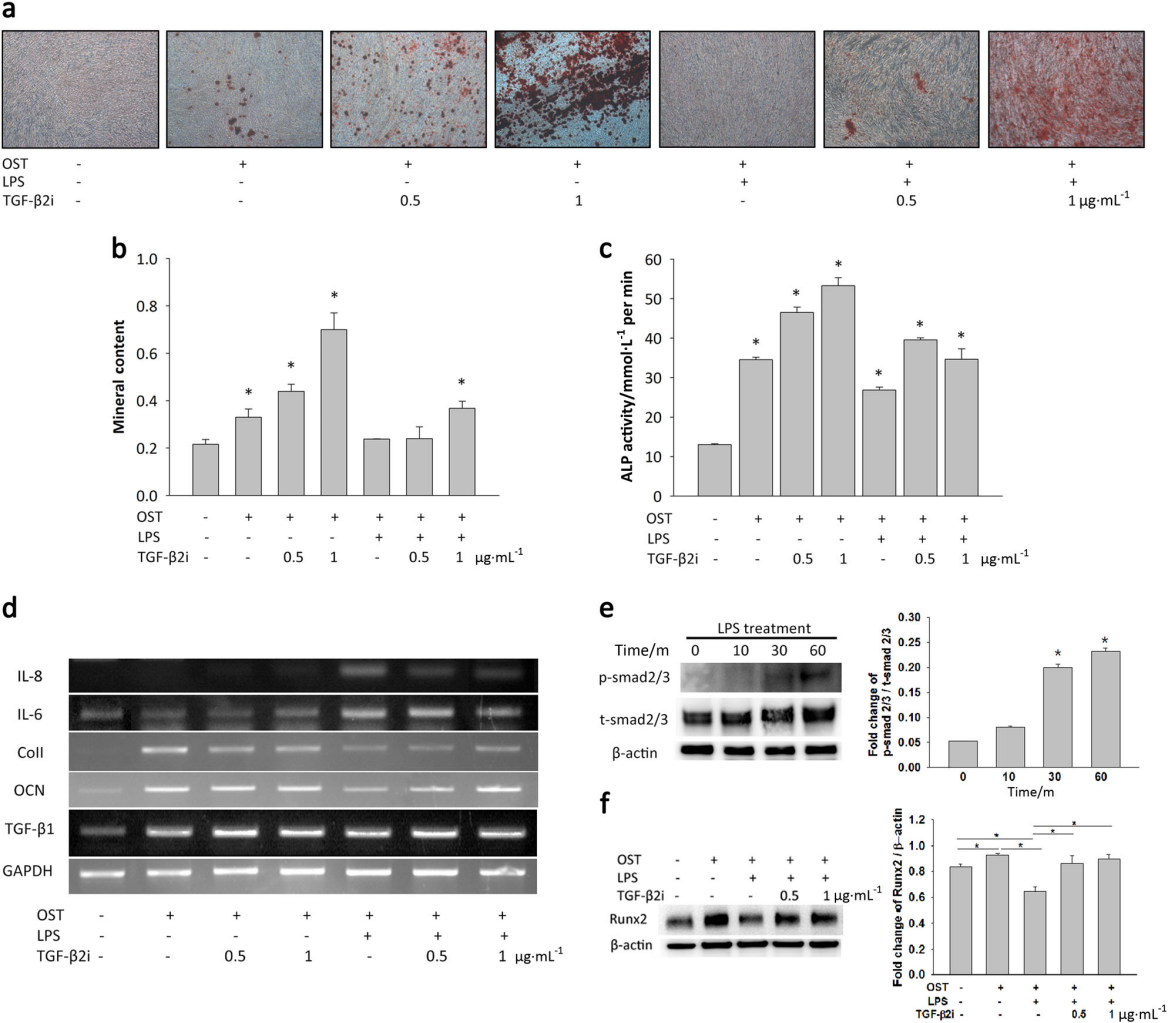
Inhibition of TGF-β2 overcomes the downregulation of bone formation caused by LPS. **a**, **b** On day 28, alizarin red S solution was used to stain calcium deposits in cultures treated with TGF-β2 inhibitor and LPS. The dissolved mineral content of the medium decreased after LPS treatment. However, treatment with 1 μg**·**mL^−1^ TGF-β2 inhibitor neutralised the TGF-β2 secreted by LPS treatment. Interestingly, inhibition of TGF-β2 increased the osteogenic differentiation of DFSCs. **c** The results of ALPase activity measurements also supported the conclusion that inhibition of TGF-β2 increased the early stage of osteogenesis in DFSCs. **d** When the TGF-β2 secreted during LPS-induced inflammation was neutralised, the levels of the pro-inflammatory cytokines IL-6 and IL-8 decreased. In contrast, osteocalcin (OCN) and type 1 collagen (Col1) expression increased after treatment of the cells with TGF-β2 inhibitor during LPS-induced inflammation. **e** Treatment with 100 ng**·**mL^−1^ LPS for 30 min activated smad2/3 signalling. **f** DFSCs activated with LPS for 30 min were treated with 0.5 μg**·**mL^−1^ TGF-β2 inhibitor for 7 days during osteogenic differentiation. In the presence of a TGF-β2 inhibitor, Runx2 expression was overcome. The data are presented as the mean ± SD. * *P* < 0.05 (*n* = 3)

To demonstrate that TGF-β2 exerts a strong influence on osteogenic differentiation, TGF-β2 inhibitors were used to prevent the action of TGF-β2 in the inflammatory environment. When TGF-β2 action is inhibited, DFSCs can differentiate into osteogenic tissues. The alizarin red S staining results (Fig. 6a, b) showed that LPS treatment suppressed osteogenic differentiation and that treatment with TGF-β2 inhibitors overcame the downregulation of osteogenic differentiation. The ALP activity of DFSCs treated with 0.5 or 1 μg**·**mL^−1^ TGF-β2 inhibitors was increased through suppression of TGF-β2 regulation (Fig. 6c). These results showed that calcium deposition by DFSCs increased after inhibition of TGF-β2.

During osteogenic differentiation in the presence of LPS, treatment of the cells with TGF-β2 inhibitors at concentrations of 0.5 and 1 μg**·**mL^−1^ overcame the suppression of osteogenesis by TGF-β2. The expression of the type I collagen (ColI) and osteocalcin (OCN) genes showed patterns similar to those observed for ALP activity and alizarin red S staining (Fig. 6d). After treatment with TGF-β2 inhibitors at 0.5 or 1 ng**·**mL^−1^, downregulation of the expression of the gene encoding OCN increased. However, neither the ColI nor the OCN gene showed a change in expression after treatment of the cells TGF-β2 inhibitors at 0.5 or 1 ng**·**mL^−1^. Additionally, TGF-β1 gene expression did not change after treatment of the cells with TGF-β2 inhibitors. Interestingly, IL-8 and IL-6 levels were increased in the presence of LPS and decreased in the presence of TGF-β2 inhibitors. Additionally, smad2/3 of DFSCs underwent phosphorylation during LPS treatment in a time-dependent manner. After treatment of DFSCs for 30 min with LPS, smad2/3 signalling was triggered (Fig. 6e). In the presence of TGF-β2 inhibitors during osteogenic differentiation, Runx2 expression was increased; low levels of RUNX2 were observed in cells treated with TGF-β2 inhibitors alone (Fig. 6f).

**Fig. 7:**
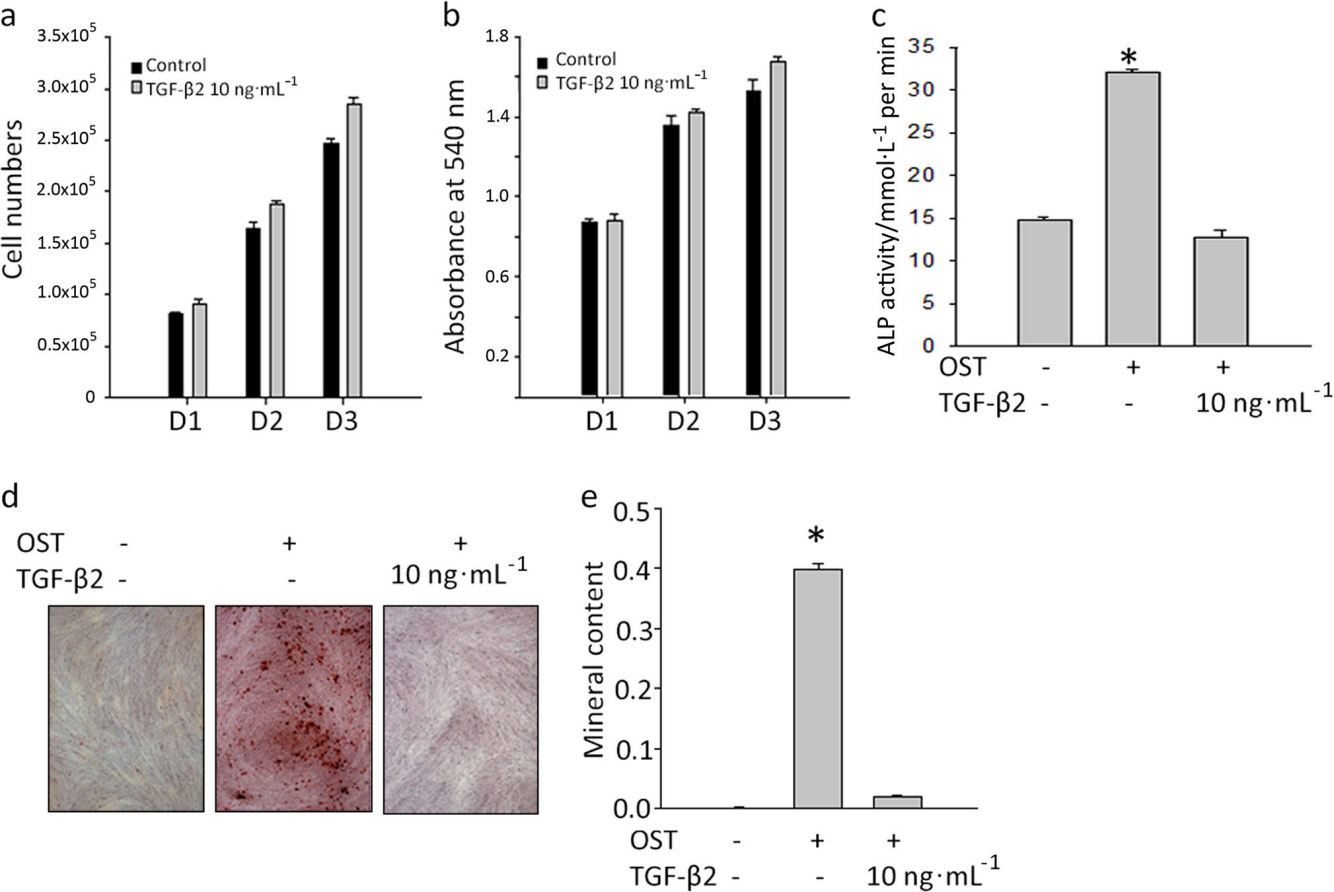
TGF-β2 affects proliferation and osteogenic differentiation. **a**, **b** TGF-β2 at 10 ng**·**mL^−1^ increased cell proliferation at days 1, 2 and 3. **c** The early osteogenic differentiation of DFSCs detected by ALP activity at day 7 was inhibited by TGF-β2 treatment. **d**, **e** Alizarin red S staining showed that 10 ng**·**mL^−1^ TGF-β2 inhibited the osteogenic differentiation of the cells. The data are presented as the mean ± SD. * *P* < 0.05 (*n* = 3)

To demonstrate the effects of TGF-β2 on DFSCs in the absence of an inflammatory environment, the cells were treated with 10 ng**·**mL^−1^ TGF-β2. The results of cell counting and MTT assays indicated that this treatment caused a slight increase in the proliferation rate of the DFSCs on days 1, 2 and 3 (Fig. 7a, b). Additionally, ALP activity and alizarin red S staining indicated that TGF-β2 inhibited the osteogenic differentiation of the cells (Fig. 7c–e). Overall, these data indicate that LPS-treated DFSCs display suppressed osteogenic differentiation and increased TGF-β2 levels.

## Discussion

Dental follicle tissue is easily obtained from developing teeth, especially wisdom teeth, during third molar extraction in adolescent patients. DFSCs have been suggested as a possible candidate for promoting the regeneration of dental tissue that has been destroyed by oral disease. In the presence of certain pathogens, the development of dental stem cells is inhibited; as a result, bone formation is suppressed. Although it is known that LPS-induced inflammation results in aberrantly low levels of bone formation, the interactions between inflammation and bone formation by MSCs are still unclear. In this study, we demonstrated that TGF-β2 triggered by inflammation mediates the inhibition of osteogenic differentiation.

Comparison of DFSCs in normal and inflamed tissues showed differences in their proliferation and in their osteogenic differentiation. Interestingly, although CFU-forming assays and the calculation of population doubling times showed that inflamed DFSCs have higher proliferative efficiency, highly proliferative inflamed DFSCs showed low levels of osteogenic differentiation. The gene expression of osteocalcin in inflamed DFSCs decreased significantly. Additionally, ALP activity measurements showed that inflammatory conditions suppressed the early stage of osteogenic differentiation in DFSCs. The results of in vivo transplantation experiments indicated that normal DFSCs show higher levels of bone formation than inflamed DFSCs. Our results show similarity to the results obtained previously on suppressive bone formation in models of osteoporosis and periodontitis.^35,36^ The use of animal models involving inflammation has shown that inflammatory cytokines can inhibit cell differentiation. However, previous work has not defined the underlying mechanisms through which inflammatory cytokines affect osteogenic differentiation.

To clarify the differences between normal and inflamed DFSCs, the proteins in each sample were analysed by LC-MS/MS. The results indicated that TGF-β1 and TGF-β2 were inversely expressed in normal and inflamed DFSCs during osteogenic differentiation. In particular, inflamed DFSCs expressed significantly higher levels of TGF-β2 than normal DFSCs. BMP-2, a member of the TGF-β superfamily, is well known to be involved in the regeneration of bone. TGF-β1 is also well known as an anti-inflammatory cytokine that inhibits the immune functions of T cells, B cells, NK cells and macrophages.^6,37^ Intriguingly, TGF-β1 secreted by MSCs is important in the mediation of T cell inhibition. However, unlike the well-studied anti-inflammatory regulator TGF-β1, the role of TGF-β2 gene expression in inflammation, especially in inflammation accompanying osteogenesis, has not been well studied. Few studies of the interactions of inflammation with TGF-β involving treatment of mouse MSCs with TGF-β1 and TGF-β2 have been performed.^37^ When the immune reaction was triggered by IFN-γ and TNF-α, treatment of mouse MSCs with TGF-β1 decreased iNOS expression, a marker of inflammation, in a dose-dependent manner. In contrast, iNOS expression was downregulated by TGF-β2 treatment. This is consistent with our results showing that inflamed DFSCs express higher levels of TGF-β2.

To mimic the local inflammatory environment and create conditions similar to those that exist in inflamed tissue, DFSCs were treated with LPS. LPS is a key factor regulating the inflammatory response and differentiation of MSCs. Some reports have suggested that LPS inhibits the osteogenic differentiation of MSCs via MAPK signalling.^31,34^ Unlike other orally derived stem cells, in previous studies DFSCs were only stimulated with LPS from Escherichia coli.^26^ In our study, the level of nitric oxide in the culture supernatant of DFSCs that received 48 h LPS treatment increased in a concentration-dependent manner. The results of NO assays were consistent with those obtained by the measurement of the levels of the inflammatory markers IL-6 and IL-8 using PCR and ELISA. The PCR data showed that the expression of IL-6 and IL-8 by LPS-treated DFSCs was slightly elevated and that there was little or no change in the expression of TGF-β1 and TGF-β2 in cells in conditional media. The protein levels of IL-6 and IL-8 were also high after LPS treatment. However, no change in the viability of the SFSCs was observed after treatment with 100 ng**·**mL^−1^ LPS. During osteogenic differentiation of LPS-treated cells, the inflammatory cytokines IL-6 and IL-8 were expressed. LPS inhibited extracellular calcium deposition during the osteogenic differentiation of DFSCs. In addition, osteocalcin expression was inhibited by LPS treatment. These findings imply that inflammation, which inevitably occurs during bone healing, may be the major cause of low osteogenic differentiation. Interestingly, TGF-β1 expression was downregulated and TGF-β2 expression was upregulated during the osteogenic differentiation of LPS-treated cells. The higher expression of TGF-β2 in LPS-treated DFSCs confirmed the similar trend observed in inflamed DFSCs.

TGF-β2 inhibitors were applied to determine whether they could neutralise the effects of LPS-triggered inflammation, which upregulates TGF-β2 expression, on bone metabolism. The results of alizarin red S staining and ALP activity measurements demonstrated that the suppression of TGF-β2 activity overcame the inhibition of osteogenic differentiation by LPS. In the presence of 1 μg**·**mL^−1^ TGF-β2 inhibitor, calcium deposition and ALP activity were increased compared to that in cells undergoing LPS-treated osteogenic differentiation. Interestingly, the increased expression of IL-6 and IL-8 triggered by LPS was suppressed by TGF-β2 inhibition. On the other hand, the levels of the osteogenic markers Col1 and OCN increased after TGF-β2 inhibition, and inhibition of TGF-β2 increased the gene expression of TGF-β1. These results indicate that inhibition of TGF-β2 suppresses the inflammatory reaction and results in an increase in bone formation by competing with TGF-β1. These results indicate that TGF-β2 is a key factor controlling both osteogenesis and inflammatory reactions.

Smad2/3 is a key regulator in the control of both inflammation and osteogenesis. Previous research using Smad2/3 knockout mice indicated that Smad2/3 is the regulator that controls the inflammation.^38^ In Smad2/3 double knockout mice, fatal inflammation was observed.^39^ T cell-specific Smad signalling is essential for iTreg induction and Th suppression.^8^ Previous research demonstrated that TGF-β2 operates through a distinct mechanism in which the type III receptor, betaglycan, functions as a co-receptor for efficient binding, in contrast to TGF-β1/3, which acts by direct binding to type I and II receptors.^40,41^ Both TGF-β1/3 and TGF-β2 act through Smad-dependent signalling. In our study, Smad2/3 was phosphorylated after treatment of DFSCs with LPS for 30 min. This shows that MSCs were also stimulated by LPS to trigger the immune reaction. Additionally, the inhibition of TGF-β2 may activate Smad2/3 signalling, thereby increasing TGF-β1 expression. Inhibition of TGF-β2 increased the gene expression of TGF-β1. Therefore, the activation of Smad2/3 by elevated TGF-β2 levels in inflammation could affect osteogenic differentiation.

A major issue that was raised by these studies and remained unresolved is whether smad2/3 is activated by TGF-β1 or TGF-β2. In DFSCs with high levels of expression of TGF-β2 during osteogenesis, TGF-β1 expression was downregulated. Because of the relationship of TGF-β1 to osteogenesis, the inhibition of TGF-β1 production has been considered a possible explanation for the impaired osteogenesis observed in LPS-treated or inflamed DFSCs. However, the results of his study, in which inflamed DFSCs and LPS-triggered DFSCs were examined, indicate that TGF-β2 is a critical factor controlling the effect of inflammation on bone formation.

In conclusion, TGF-β2 is a key factor in understanding why lower levels of bone formation occur during inflammation. TGF-β2 can be used in two ways to explain the mechanisms involved in the relationship between bone formation and inflammation. By sharing the Smad2/3 signalling pathway, the increased TGF-β2 levels produced by inflammation affect the expression of osteogenesis-related genes such as osteocalcin, type I collagen and RUNX2. The results of this study suggest that the interactions between inflammation and bone formation are regulated in a complex manner by TGF-β2. In the future, the control of TGF-β2 levels during the therapeutic use of DFSCs in periodontal disease to regenerate bone formation could elicit low levels of inflammatory reactions.

## Materials and methods

### Primary cell cultures

Human normal (*n* = 6, male/female, age 15–21) and inflamed primary dental follicles (*n* = 1, female, age 18) were collected at the Department of Oral and Maxillofacial Surgery of Seoul National University Dental Hospital. The experiments were performed according to a protocol that was approved by the Institutional Review Board of the Seoul National University School of Dentistry (IRB No. S-D20080009). Cell isolation was performed as described previously with modifications.^42^ The collected DFSCs were incubated at 37 °C for 1 h in 3 mg**·**mL^−1^ type 1 collagenase (BioBasic INC., Toronto, ON, Canada) and 4 mg**·**mL^−1^ dispase II (Gibco-BRL, Waltham, MA, USA). The isolated MSCs were cultured in alpha minimum essential medium (α-MEM; Gibco-BRL) containing l-ascorbic acid (100 μmol**·**L^−1^, BioBasic INC.), l-glutamine (2 mmol**·**L^−1^, Gibco-BRL), penicillin (100 units per mL, Gibco-BRL), streptomycin (100 μg**·**mL^−1^, Gibco-BRL), amphotericin B (0.25 μg**·**mL^−1^, Gibco-BRL), and 15% foetal bovine serum (FBS; Equitech-Bio Inc., Kerrville, TX, USA). The cells were incubated at 37 °C in a humidified 5% CO_2_ atmosphere, and the medium was changed every 2–3 days. DFSCs from early passages (1–6) were used in these experiments.

### Proliferation and cell viability assays

Normal and inflamed DFSCs of each passage were cultured for 3 days. For colony-forming assay, 500 cells were seeded; after 10 days of culture, the cells were fixed with 4% formaldehyde (Sigma-Aldrich Co.) and stained with 1% crystal violet (Sigma-Aldrich Co.). Colonies consisting of more than 50 cells were counted at passages 2 and 4. CFE was calculated as a percentage of the number of seeded cells. To measure the cumulative population doubling time, normal and inflamed dental follicle stem cells were counted on days 3, 6, 9, 13, 16 and 19. Cell viability was measured using a 3-(4,5-dimethylthiazol-2-yl)−2,5-diphenyltetrazolium bromide (MTT) assay. The optical density of formazan crystals dissolved in DMSO was read at 540 nm.

### In vitro osteogenic differentiation

When the cells were 40–50% confluent, the culture medium was replaced with α-MEM containing dexamethasone (10 nmol**·**L^−1^, Sigma-Aldrich Co., St. Louis, MO, USA), glycerol phosphate (5 mmol**·**L^−1^, Sigma-Aldrich), penicillin (100 units per mL, Gibco-BRL), streptomycin (100 μg**·**mL^−1^, Gibco-BRL), amphotericin B (0.25 μg**·**mL^−1^, Gibco-BRL), l-ascorbic acid (100 μmol**·**L^−1^, BioBasic Inc.) and 10% FBS (Equitech-Bio Inc.) for osteogenic differentiation of the cells in the presence or absence of 100 ng**·**mL^−1^ LPS and TGF-β2 inhibitor (R&D Systems, Minneapolis, MN, USA). On day 7, ALP activity was measured using a QuantiChrom assay kit (BioAssay Systems, Hayward, CA, USA) according to the instructions provided by the manufacturer. 14–28 days after induction, the cells were stained with 40 mmol**·**L^−1^ alizarin red S solution (pH 4.2, Sigma-Aldrich) for 15 min to visualise the calcium accumulation in mineralised cells. Quantification of alizarin red S was performed by destaining with a solution of 20% methanol and 10% acetic acid. The optical density of the solvent was read at an absorbance of 450 nm.

### RT-PCR and real-time PCR

Total RNA collected using an RNA mini kit (Ambion, Carlsbad, CA, USA) was reverse-transcribed to complementary DNA using the SuperScript III First-Strand Synthesis Systems kit (Invitrogen, Carlsbad, CA, USA). A real-time PCR System 7500 (Applied Biosystems, Foster City, CA, USA) was used to measure mRNA expression using primers and probes for glyceraldehyde-3-phosphate dehydrogenase (GAPDH, Hs99999905_m1) as an endogenous control, TNF-α (Hs01113624_g1), IL-6 (Hs00985639_m1), IL-8 (Hs00174103_m1), osteocalcin (Hs01587814_g1), ALP (Hs01029144_m1), and type I collagen (Hs00164004_m1). mRNA expression was examined using specific primers for IL-1β (forward, 5'-TCATTGCTCAAGTGTCTGAAGC-3'; reverse, 5'-TGGTCGGAGATTCGTAGC-3'), IL-8 (forward, 5'-ACTGAGAGTGATTGAGAGTGGAC-3'; reverse, 5'-AACCCTCTGCACCCAGTTTTC-3'), IL-6 (forward, 5'-GAA AGCAGCAAAGAGGCACT-3'; reverse, 5'-TTTCACCAGGCAAGTCTCCT-3'), stathmin (forward, 5'-ACTGCCTGTCGCTTGTCT; reverse, 5'-GTCTCGTCAGCAGGGTCT-3'), spondin-2 (forward, 5'-CGGCCAAATACAGCATCACC-3'; reverse, 5'-CCCAGCAGCGAAGACCACT-3'), layilin (forward, 5'-AGGAGTAAGGAGTCTGGATGGGTG-3'; reverse, 5'-GGATGACTGGCTGGGATAAAGGA-3'), TGF-β1 (forward, 5'-GGACACCAACTATTGCTTCAG-3'; reverse, 5'-TCCAGGCTCCAAATGTAGG-3'), TGF-β2 (forward, 5'-GGCTCAGTGGGCAGCTTGT-3'; reverse, 5'-GCTCAATCCGTTGTTCAGGC-3'), and GAPDH (forward, 5'-AGCCGCATCTTCTTTTGCGTC-3'; reverse, 5'-TCATATTTGGCAGGTTTTTCT-3').

### In vivo transplantation and Immunohistochemistry

The care, maintenance, and treatment of animals in these experiments followed protocols approved by the Institutional Animal Care Committee of Seoul National University (SNU-140501-9-1). Approximately 5 × 10^6^ normal and inflamed DFSCs were implanted into the dorsal skin of immunocompromised nude mice (NIH-bg-nu-xid, Harlan Sprague Dawley, Indianapolis, IN, USA) together with 40 mg of hydroxyapatite/tricalcium phosphate particles (HA/TCP, Zimmer, Warsaw, IN, USA). The transplants were harvested and fixed with 4% paraformaldehyde. The fixed transplants were decalcified with 10% EDTA (pH 8.0, Sigma-Aldrich) and embedded in paraffin. Haematoxylin and eosin (H&E) staining was performed on tissue sections. For immunohistochemical (IHC) staining, mouse anti-human mitochondria antibody (1:200, Chemicon, Temecula, CA), mouse anti-human osteocalcin antibody (1:100, Abcam, Cambridge, UK) and rabbit anti-human collagen type 1 (1:800, Abcam) were used followed by detection with an anti-mouse/rabbit HRP/DAB detection kit (Abcam).

### Protein profile analysis and mass spectrometry

Normal and inflamed DFSCs were cultured for 24 h, and the culture supernatants were collected. The proteins present in the cell culture supernatants were purified and concentrated using centrifugal filter devices (Merck, Billerica, MA, USA). Aliquots (25 μg) of the concentrated proteins were analysed and compared using 12% sodium dodecyl sulphate-polyacrylamide gel electrophoresis (SDS-PAGE) followed by staining with Coomassie brilliant blue (CBB). Selected bands from 1-dimensional gel electrophoresis (1-DE) were digested with trypsin and analysed by LC-MS/MS. All tandem mass spectra were searched against the NCBI database using the Mascot search engine (Matrix Science, London, U.K.). Peptides with Mascot scores of 30 or above were identified.

### Nitric oxide (NO) assay

To mimic the inflammatory environment in dental follicle tissue, DFSCs were seeded and cultured in the presence of *P.g.-*LPS (055:B5, Sigma-Aldrich Co., St. Louis, MO, USA). The culture supernatants were collected after incubation of the cells with 10, 100 and 1000 ng**·**mL^−1^ LPS for 24 or 48 h. Nitric oxide production levels were measured using a Quantichrom NO assay kit (BioAssay Systems) according to the manufacturer’s instructions. The absorbance was read at 540 nm.

### ELISA

Supernatants from DFSCs treated with 10, 100, 1000 ng**·**mL^−1^ LPS for 24 and 48 h were collected, and the concentrations of IL-6 and IL-8 in the cell culture supernatants were measured. Human IL-6 and IL-8 were measured using Quantikine ELISA kits (RnD Systems, Minneapolis, MN, USA). The absorbance was read at 450 nm.

### Western blot

DFSCs were seeded onto six-well plates at 2 × 10^5^ cells per well. After overnight starvation, the cells were treated with 0.1 μg**·**mL^−1^ LPS for 30 min in the presence or absence of 0.5 or 1 μg**·**mL^−1^ TGF-β2 inhibitor. Cell extracts were prepared in lysis buffer (Invitrogen) containing protease inhibitors and 1 mmol**·**L^−1^ phenylmethanesulphonyl fluoride at 5, 10, 15, 30, 60, and 120 min. The proteins in each sample were separated, transferred to PVDF membranes, and detected using antibodies against p-smad 2/3, smad 2/3, and β-actin (1:1 000; Cell Signalling Technologies, Boston, MA, USA) and an HRP-linked secondary antibody (1:1 000). The immunoblots were visualised using an HRP chemiluminescent detection kit (SurModics, Eden Prairie, MN, USA) and measured using a MicroChemi analyser (DNR Bio-image Analyzer).

### Statistical analysis

The data are presented as the mean ± standard deviaitons (SD) of the values obtained in experiments performed at least in triplicate. Statistically significant differences between groups were assessed using Student’s t-test and one-way ANOVA followed by Tukey’s HSD test in SPSS 22 (SPSS Inc., Chicago, IL, USA). *P* < 0.05 was considered to indicate statistical significance.

## Electronic supplementary material

Cell viability by LPS treatment
