# Supplementary figures and images for: TGF-β2 downregulates osteogenesis under inflammatory conditions in dental follicle stem cells
Source: Int J Oral Sci. 2018 Oct 9;10(3):29. doi: 10.1038/s41368-018-0028-8 (PMC6175959; doi:10.1038/s41368-018-0028-8)

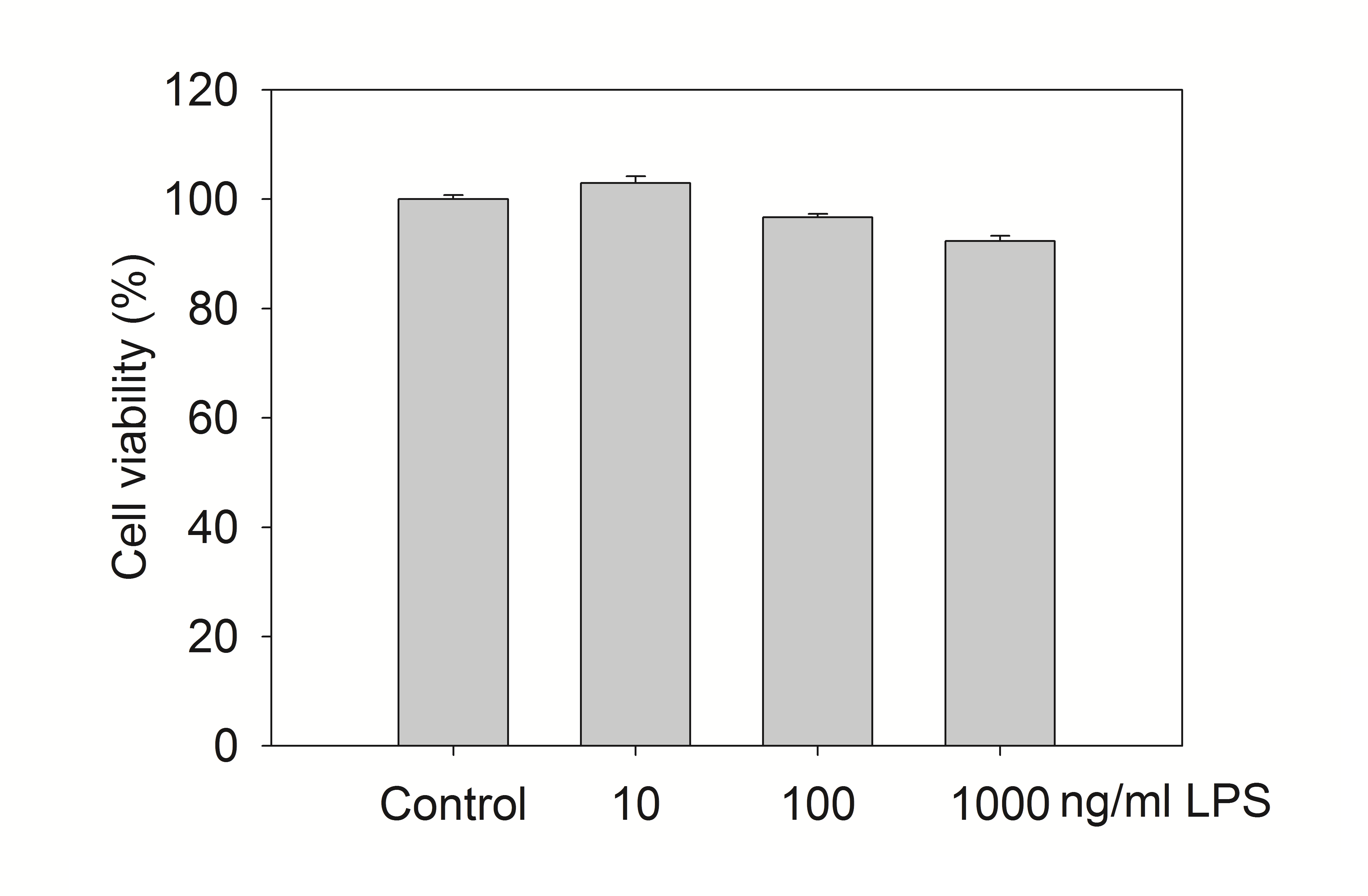

Supplement: Supplementary file 1 — Cell viability by LPS treatment [file 41368_2018_28_MOESM1_ESM.tif]
